# The impact of professional identity on nurses’ mental health status after the COVID-19 public health emergency: a cross-sectional survey

**DOI:** 10.3389/fpubh.2025.1678636

**Published:** 2025-11-17

**Authors:** Nan Xie, Liqun Zou, Zhenfei Yuan

**Affiliations:** 1 Department of Emergency Medicine, West China Hospital, Sichuan University/West China School of Nursing, Sichuan University, Chengdu, China; 2 Disaster Medical Center, Sichuan University, Chengdu, China

**Keywords:** China, nursing staff, professional identity, anxiety, depression, sleep quality, COVID-19, cross-sectional study

## Abstract

**Aim:**

The purpose of this study was to explore the correlation between the mental health status and professional identity of Chinese nurses after the COVID-19 public health emergency.

**Method:**

An e-questionnaire survey was designed, and nurses from 4 Chinese hospitals were included in our study. The questionnaires were used to measure nurses’ professional identity, mental health, and sleep quality. Logistic regression analyses were used to explore their relationships.

**Results:**

The multiple logistic regression analysis suggested that medical assistance, PSQI score, and level of professional identity were independent risk factors for anxiety, and the results of multiple logistic regression analysis suggested that a medium level of professional identity and the PSQI score were independent factors for depression.

**Discussion:**

The results show that nurses’ professional identity is negatively related to anxiety and depression after experiencing the COVID-19 public health emergency.

**Implications for practice:**

After the COVID-19 public health emergency, managers urgently need to pay attention to nurses’ mental health issues, which may help improve nurses’ professional identity, increase their work enthusiasm, and benefit their career development.

## Introduction

The COVID-19 pandemic, declared a Public Health Emergency of International Concern (PHEIC) by the World Health Organization, serves as a critical context for this study, providing a unique lens to examine the interplay between professional identity and mental health among nurses during a global health crisis. It is a special event of disease spread that will cause health, economic and social consequences and require coordinated actions by the government and society. Nurses play a key role in health caring, with more than half of health workers still on the clinical frontline, which increases the mental health risks in health workers (1). However, nurses are also considered assistants to doctors, which results in a poor impression and low social status of nursing work in the modern social environment (2). It has been shown that the COVID-19 public health emergency has increased heavy pressure on nurses, which has caused higher turnover and reduced the level of nurses’ professional identity level. Professional identity is the best predictor of the departure rate from nursing (3). The aging population is a severe issue, and nurses are shorthanded in China. These reasons lead to the heavy shortage of nursing staff (4).

Professional identity was defined as “one’s professional self-concept based on attributes, beliefs, values, motives, and experience” (5, 6), which plays a crucial role in nurses, and it has been changing constantly in nursing careers (7). One study reported that nurses are more likely to be mobile during major public health emergency (8). Previous research argued that nurses’ high level of professional identity can improve clinical performance (9), increase job retention (10, 11), and provide high-quality services (12).

Mental health is a multidimensional construct that includes emotional, cognitive, and behavioral well-being, and its disturbances commonly manifest as anxiety, depression, and sleep disorders in nurses after major public health events. Anxiety and depression are among the most prevalent and well-validated indicators of post-crisis psychological burden in nursing populations; therefore, the GAD-7 and SDS were selected in this study as standardized and widely used tools. Furthermore, a growing body of longitudinal and review evidence indicates a strong bidirectional relationship between sleep disturbance and psychological distress (13, 14): insomnia not only commonly co-occurs with anxiety and depression, but longitudinal studies and meta-analyses show that insomnia symptoms predict future onset of depressive and anxiety symptoms. For example, Baglioni et al. (15) reported that insomnia roughly doubles the risk of later depression in prospective studies, and mechanistic reviews identify disrupted sleep and circadian functioning as core processes in mood and anxiety disorders (16). Therefore, we included the PSQI to assess sleep quality as an integral dimension of mental health rather than limiting mental health to emotional symptoms alone.

Furthermore, sleep disturbance has a strong bidirectional relationship with psychological distress, and insomnia is both a predictor and a consequence of anxiety and depression. For this reason, the PSQI was included to assess sleep quality as an integral dimension of mental health, rather than limiting mental health to emotional symptoms alone.

This study draws upon Social Identity Theory and Professional Identity Formation Theory to understand the relationship between professional identity and mental health (6). According to Social Identity Theory (17), individuals derive part of their self-concept from their membership in social and professional groups. A strong and positive professional identity contributes to psychological resilience, helping individuals maintain a coherent and valued sense of self during times of stress or crisis. Conversely, when professional identity is weakened or threatened—as during public health emergencies—individuals may experience increased psychological distress such as anxiety and depression. By applying this theoretical lens, we examine how variations in nurses’ professional identity relate to their mental health status following a major public health crisis like COVID-19.

Although prior studies have examined links between professional identity and mental health in nursing populations (18), relatively few quantitative studies have examined this relationship specifically in the aftermath of a major public health emergency. The present study therefore aimed to examine the association between nurses’ professional identity and symptoms of anxiety and depression following the COVID-19 public health emergency. Our primary objective was to test whether professional identity scores were associated with concurrent symptoms of anxiety and depression in a sample of Chinese nurses surveyed in February 2023.

## Methods

### Study design and population

A cross-sectional survey was conducted among registered nurses working in four hospitals in Sichuan Province, China between February 2 and February 28, 2023.

Inclusion criteria were: (1) registered nurses; (2) participated in clinical work during the COVID-19 public health emergency; (3) provided informed consent. Exclusion criteria were: non-nursing staff and questionnaires with incomplete key variables. All nurses in this study had direct pandemic-related work experience; therefore, no unexposed nurses were included. Sample size adequacy was evaluated using G*Power, and our final sample exceeded the minimum requirement. The questionnaire did not set an answer-time exclusion rule because the survey content was straightforward, and completion time was expected to vary across individuals. Working-year differences were analyzed in univariate and adjusted models to compare mental health between nurse groups with different experience levels.

### Recruitment and data collection

Recruitment was conducted using internal hospital communication channels (email or WeChat groups). The survey was hosted on Wenjuanxing (www.wjx.cn), a widely used online survey tool in China similar to Qualtrics or SurveyMonkey, providing secure and user-friendly interfaces for data collection. All participants were voluntary and anonymous. The questionnaire could be completed only once per participant using the same Internet Protocol (IP) address to prevent duplicate submissions; however, IP addresses were not stored in the exported dataset, preserving anonymity. All participants could withdraw at any time without giving any reason. There were minimal missing values (<2% per item), and cases with incomplete key variables were excluded from the final analysis (*n* = 5).

### Sample size calculation

*A priori* sample size calculation was performed using G*Power 3.1.9.7 for logistic regression. We designated the primary outcome as presence of anxiety (GAD-7 ≥ 5). This choice was made because the GAD-7 is a validated, brief screening instrument widely used in healthcare worker studies during the COVID-19 pandemic (19), and a cut-point of 5 is commonly used to denote presence of any clinically relevant anxiety symptoms (mild or greater) in population and occupational studies (20), thereby capturing a clinically meaningful burden while maximizing the observed event rate for regression modeling. Using GAD-7 ≥ 5 as the binary outcome also aligns with prior COVID-era HCW studies that used the same threshold for reporting anxiety prevalence (1). Assuming a medium effect size approximated by an odds ratio of 1.5 for a binary predictor, *α* = 0.05, power (1 − *β*) = 0.80, and inclusion of up to 10 candidate predictors in multiple models, the minimum required sample size was estimated at 142. Our final sample of 253 participants exceeds this requirement. We acknowledge that convenience sampling limits generalizability; therefore, the sample size calculation is reported for transparency rather than implying population representativeness.

### Ethics statement

This study received unified ethics approval from the Ethics Committee of West China Hospital (No. 2023/177), and the approval covered all participating sub-centers. Written informed consent was obtained electronically from all participants prior to participation. This study was performed in accordance with Strengthening the Reporting of Observational Studies in Epidemiology (STROBE) guidelines.

### Measures

The e-questionnaire comprised a demographic questionnaire, Nurses’ Professional Identity Scale, Generalized Anxiety Disorder (GAD-7), Self-rating Depression Scale (SDS), and Pittsburgh Sleep Quality Index (PSQI).

### Demographics

Participants completed a demographic questionnaire including age, gender, education level, professional title, marital status, child status, years of working, medical assistance experience (defined as deployment to support other regions or hospitals during the COVID-19 response), department, working on the clinical frontline and underlying medical conditions.

### Professional identity scale for nurses

The Professional Identity Scale for Nurses developed by Liu et al. (21) consists of 30 items grouped into five dimensions (professional identity evaluation, professional social support, professional social proficiency, dealing with professional frustration, and professional self-reflection). Each item is rated on a 5-point Likert scale (1 = strongly disagree to 5 = strongly agree), with higher scores indicating a stronger professional identity. The total score ranges from 30 to 150. A professional identity norm (21) classifies total scores of ≤90 as low, 91–120 as medium, and > 120 as high professional identity. In this study, we re-checked all item codings (including reverse-scored items) and recalculated reliability. The total-scale Cronbach’s *α* was 0.938, indicating excellent internal consistency. Subscale α coefficients were professional identity evaluation = 0.913, professional social support = 0.876, professional social proficiency = 0.892, dealing with professional frustration = 0.905, and professional self-reflection = 0.881. These values are consistent with prior Chinese nurse validation studies (21).

### Generalized anxiety disorder 7-item

The GAD-7 is a 7-item self-report measure of generalized anxiety severity developed by Spitzer et al. (20). Each item is rated 0 (not at all) to 3 (nearly every day), yielding a total score of 0–21; cut-off values of 5, 10, and 15 represent mild, moderate, and severe anxiety, respectively. The Chinese version has demonstrated strong reliability and validity (22). In our sample, Cronbach’s *α* was 0.915.

### Self-rating depression scale

The Zung SDS (23) comprises 20 items, each rated 1–4 (a little of the time to most of the time). Ten items are reverse scored. The raw total (20–80) is multiplied by 1.25 to obtain the standard score (25–100); a standard score ≥ 50 indicates possible depressive symptoms. In our sample, Cronbach’s *α* was 0.874.

### Pittsburgh sleep quality index

The Chinese version of the PSQI (20) scale was used to evaluate anxiety in the previous month, and it has been most widely used to evaluate sleep quality in many studies. It contains 19 self-reported items and is divided into seven components. Each component was scored from 0 = No difficulty to 3 = Severe difficulty; component scores were summed to produce the global PSQI (0–21), and a total score ≥ 7 indicates poor sleep quality (24). Higher scores indicate poor sleep quality. In this study, Cronbach’s *α* was 0.821.

### Statistical analysis

IBM SPSS Statistics for Windows, Version 25.0, was used for data analyses. Continuous variables are presented as mean ± SD or median [IQR] depending on distribution. Categorical variables are presented as n (%). Group comparisons used Student’s t-test or Mann–Whitney U test for continuous variables and Chi-square or Fisher’s exact test for categorical variables. Spearman rank correlation was used to examine the association between professional identity and GAD-7/SDS scores.

To identify independent factors associated with anxiety and depression, multiple logistic regression analyses were conducted. Candidate variables with *p* < 0.05 in univariate analyses were entered into multiple logistic regression models. Multicollinearity was assessed using VIF (values >5 considered indicative of problematic collinearity). Backward stepwise selection (entry *p* = 0.05, removal *p* = 0.10) was used to derive the final model. Model fit was evaluated with the Hosmer-Lemeshow test and receiver operating characteristic (ROC) AUC. All tests were two-tailed and *p* < 0.05 was considered statistically significant.

## Results

### Characterization and distribution of anxiety and depression

As shown in Table 1, a total of 136 nurses had anxiety, and while 117 did not. There were significant differences in job titles (*p* = 0.009), marital status (*p* = 0.080), years of working (*p* = 0.012), medical assistance (*p* = 0.006), working on the clinical frontline (*p* = 0.026), PSQI score (*p* < 0.001) and level of professional identity (*p* < 0.001) between the groups with and without anxiety. Additionally, a total of 156 nurses had depression, and while 97 did not. Age distribution (*p* = 0.040), working on the clinical frontline (*p* = 0.017), PSQI score (*p* < 0.001) and the level of professional identity (*p* = 0.004) were significantly different between the depression groups and non-depressing groups. Spearman correlation analysis showed that the scores of all dimensions and the total score of nurses’ professional identity were negatively correlated with the scores of anxiety and depression (all *p* < 0.01), indicating that higher professional identity was associated with lower anxiety and depression (Table 2).

**Table 1:** Characterization and distribution of anxiety and depression.

Parameters	Anxiety				Depression			
	Non-anxiety group	Anxiety group	*X* ^2^	*p*	Non-depression group	Depression group	*X* ^2^	*p*
Age, year								
18–45	110 (46.0%)	129 (54%)	0.840	0.772	88 (36.8%)	151 (63.2%)	4.220	0.04
46–69	7 (50%)	7 (50%)			9 (64.3%)	5 (35.7%)		
Gender								
Men	13 (59.1%)	9 (40.9%)	1.599	0.206	8 (36.4%)	14 (63.6%)	0.040	0.842
Women	104 (45.0%)	127 (55%)			89 (38.5%)	142 (61.5%)		
Education level								
Junior college	19 (59.4%)	13 (40.6%)	2.620	0.270	13 (40.6%)	19 (59.4%)	0.138	0.933
Undergraduate	95 (44.2%)	120 (55.8%)			82 (38.1%)	133 (61.9%)		
Postgraduate and above	3 (50%)	3 (50%)			2 (33.3%)	4 (66.7%)		
Title								
Junior or low	79 (53.7%)	68 (46.3%)	9.450	0.009	57 (38.8%)	90 (61.2%)	4.917	0.086
Intermedia	30 (33.3%)	60 (66.7%)			10 (33.3%)	60 (66.7)		
Senior	8 (50%)	8 (50%)			10 (62.5%)	6 (37.5%)		
Marital status								
Single	51 (55.4%)	41 (44.6%)	5.045	0.080	38 (41.3%)	54 (58.7%)	0.773	0.680
Married	64 (40.8%)	93 (59.2%)			58 (36.9%)	99 (63.1%)		
Divorced	2 (50.0%)	2 (50.0%)			1 (25%)	3 (75%)		
Child status								
Have child	56 (42.4%)	76 (57.6%)	1.621	0.203	53 (40.2%)	79 (51.3%)	0.383	0.536
No child	61 (50.4%)	60 (49.6%)			44 (36.4%)	77 (63.6%)		
Years of working								
1–5 years	48 (55.2%)	39 (44.8%)	8.796	0.012	34 (39.1%)	53 (60.9%)	1.105	0.576
6–10 years	35 (51.5%)	33 (48.5%)			29 (42.6%)	39 (57.4%)		
>10 years	34 (34.7%)	64 (65.3%)			34 (34.7%)	64 (65.3%)		
Medical assistance								
Yes	46 (37.4%)	77 (62.6%)	7.536	0.006	43 (35.0%)	80 (65.0%)	1.157	0.282
No	71 (54.6%)	59 (45.4%)			54 (41.5%)	76 (58.5%)		
Department or ward								
Emergency department	34 (44.7%)	42 (55.3%)	1.819	0.874	29 (38.2%)	47 (61.8%)	7.567	0.182
Surgical	40 (49.4%)	41 (50.6%)			30 (37%)	51 (63%)		
Internal medical	20 (41.7%)	28 (58.3%)			16 (33.3%)	32 (66.7%)		
ICU	3 (33.3%)	6 (66.7%)			1 (11.1%)	8 (88.9%)		
Anesthesia operating room	9 (50%)	9 (50%)			9 (50%)	9 (50%)		
Auxiliary clinical department	11 (52.4)	10 (47.6%)			12 (57.1%)	9 (42.9%)		
Working on the clinical frontline								
Yes	91 (43.1%)	120 (56.9%)	4.968	0.026	74 (35.1%)	137 (64.9%)	5.745	0.017
No	26 (61.9%)	16 (38.1%)			23 (54.8%)	19 (45.2%)		
Underlying medical conditions								
Yes	10 (37%)	17 (63%)	1.031	0.310	10 (37%)	17 (63%)	0.022	0.883
No	107 (47.3%)	119 (52.7%)			87 (38.5%)	139 (61.5%)		
PSQI score								
Good sleep (PSQI score <7)	44 (84.6%)	8 (15.4%)	38.765	<0.001	40 (76.9%)	12 (23.1%)	41.216	<0.001
Poor sleep (PSQI score ≥7)	73 (36.3%)	128 (63.7%)			57 (28.4%)	144 (71.6%)		
Level of professional identity								
Low	74 (38.5%)	118 (61.5%)	19.010	<0.001	64 (33.3%)	128 (66.7%)	8.443	0.004
Medium and above	43 (70.5)	18 (29.5%)			33 (54.1%)	28 (45.9%)		

**Table 2:** Correlation analysis between professional identity and anxiety/depression in nurses (r).

Correlation	Professional identity evaluation	Professional social support	Professional social proficiency	Dealing with professional frustration	Professional self-reflection	Total score
Anxiety	−0.452^**^	−0.440^**^	−0.440^**^	−0.446^**^	−0.463^**^	−0.460^**^
Depression	−0.500^**^	−0.472^**^	−0.472^**^	−0.500^**^	−0.478^**^	−0.500^**^

^**^p < 0.01.

### Professional identity and comparison with Liu’s study

The mean score of professional identity was 105.25 ± 20.32 in all included nurses. The total mean scores of this study and Liu’s study were 105.15 ± 20.32 and 96.83 ± 14.99, respectively. This study showed a medium level of professional identity, which is the same as Liu’s study. The mean score of professional identity showed a significant difference in the dimensions of professional identity evaluation (3.34 ± 0.79) compared with Liu’s study (2.88 ± 0.66), and the other four dimensions showed the same levels (as shown in Table 3).

**Table 3:** Comparison the scores on professional identity for nurse between this study and the Liu’ study.

Dimensions	This study Mean (SD)	Liu’ study Mean (SD)	t	*p*
Professional Identity evaluation	3.34 (0.79)	2.88 (0.66)		
Professional social support	3.68 (0.67)	3.58 (0.53)		
Professional social proficiency	3.36 (0.70)	3.12 (0.53)		
Dealing with professional frustration	3.67 (0.67)	3.42 (0.57)		
Professional Self-reflection	3.65 (0.78)	3.37 (0.65)		
Number	253	524		
Total score	105.25 (20.32)	96.83 (14.99)	6.505	<0.001

### Univariate and multiple logistic regression analysis of anxiety and depression

To further investigate the factors contributing to anxiety and depression, univariate and multiple logistic regression analyses were performed. In Table 4, the results of univariate analysis showed that intermediate titles (OR 2.324, 95%CI: 1.347–4.008; *p* = 0.002), married (OR 1.808, 95%CI: 1.075–3.040; *p* = 0.026), more than 10 years of working experience (OR 2.317, 95%CI: 1.281–4.192; *p* = 0.005), and poor sleep quality (OR 9.644, 95%CI: 4.306–21.598; *p* < 0.001) increased the risk of anxiety compared with junior or low titles, single, young nurses, and good sleep quality. Nurses who had medical assistance (OR 2.014, 95%CI: 1.219–3.330; *p* = 0.006) and working on the clinical frontline (OR 2.143, 95.0% CI: 1.086–4.229; *p* = 0.028) had an increased risk of anxiety compared with those who did not. A medium level of professional identity was associated with a lower risk of anxiety than a low level of professional identity (OR 0.253, 95%CI: 0.128–0.498; *p* < 0.001). In Table 5, the results of multiple analysis suggested that medical assistance (OR 2.294, 95%CI: 1.262–4.169; *p* = 0.006), PSQI score (OR 7.383, 95%CI: 3.142–17.349; *p* < 0.001), and level of professional identity (OR 0.272, 95% CI: 0.124–0.593; *p* = 0.001) were independent risk factors for anxiety.

**Table 4:** Univariate logistic regression analysis of anxiety and depression.

Parameters	Anxiety			Depression		
	*p* value	OR	95%CI	*p* value	OR	95%CI
Age, year						
46–69 vs. 18–45	0.772	0.853	0.290–2.506	0.049	0.324	0.105–0.997
Gender						
Women vs. Men	0.211	1.764	0.725–4.289	0.842	0.912	0.368–2.261
Education level						
Undergraduate vs. Junior college	0.112	1.846	0.868–3.928	0.788	1.110	0.520–2.366
Postgraduate and above vs. Junior college	0.671	1.462	0.254–8.401	0.738	1.368	0.218–8.600
Title						
Intermedia vs. Junior or low	0.002	2.324	1.347–4.008	0.399	1.267	0.731–2.195
Senior vs. Junior or low	0.776	1.162	0.414–3.261	0.075	0.380	0.131–1.102
Marital status						
Married vs. Single	0.026	1.808	1.075–3.040	0.495	1.201	0.709–2.034
Divorced vs. Single	0.831	1.244	0.168–9.215	0.524	2.111	0.211–21.076
Child status						
Have child vs. No child	0.203	1.380	0.840–2.266	0.536	0.852	0.512–1.416
Years of working						
6–10 years vs. 1–5 years	0.647	1.160	0.614–2.192	0.654	0.863	0.452–1.645
>10 years vs. 1–5 years	0.005	2.317	1.281–4.192	0.537	1.208	0.664–2.197
Medical assistance						
Yes, vs. No	0.006	2.014	1.219–3.330	0.283	1.322	0.795–2.199
Department or ward						
Surgical vs. Emergency department	0.560	0.830	0.443–1.555	0.885	1.049	0.550–2.002
Internal medical vs. Emergency department	0.737	1.133	0.546–2.353	0.587	1.234	0.578–2.633
Anesthesia operating room vs. Emergency department	0.687	0.810	0.289–2.264	0.360	0.617	0.220–1.734
ICU vs. Emergency department	0.517	1.619	0.337–6.956	0.142	4.936	0.587–41.527
Auxiliary clinical department vs. Emergency department	0.535	0.736	0.279–1.938	0.123	0.463	0.174–1.233
Working on the clinical frontline						
Yes, vs. No	0.028	2.143	1.086–4.229	0.018	2.241	1.147–4.380
Underlying medical conditions						
Yes, vs. No	0.313	1.529	0.671–3.483	0.883	1.064	0.466–2.430
PSQI score						
Poor sleep (PSQI score <7) vs. good sleep (PSQI score ≥7)	<0.001	9.644	4.306–21.598	<0.001	8.421	4.122–17.202
Level of professional identity						
Medium and above vs. low	<0.001	0.253	0.128–0.498	<0.001	0.191	0.086–0.423

**Table 5:** Multiple logistic regression analysis of anxiety/depression among nurses.

Parameters	Anxiety			Depression		
	*p* value	OR	95%CI	*p* value	OR	95%CI
Age, year						
46–69 vs. 18–45	–	–	–	0.359	0.481	0.001–2.294
Title						
Intermedia vs. Junior or low	0.302	1.558	0.671–3.616	0.736	0.772	0.171–3.477
Marital status						
Married vs. Single	0.591	1.277	0.523–3.115	–	–	–
Years of working						
>10 years vs. 1–5 years	0.358	1.719	0.541–5.460	–	–	–
Medical assistance						
Yes, vs. No	0.006	2.294	1.262–4.169			
Working on the clinical frontline						
Yes, vs. No	0.276	1.549	0.705–3.402	0.326	1.460	0.686–3.109-
PSQI score						
Poor sleep (PSQI score<7) vs. good sleep (PSQI score≥7)	<0.001	7.383	3.142–17.349	<0.001	6.838	3.260–14.324
Level of professional identity						
Medium and above vs. low	0.001	0.272	0.124–0.593	0.002	0.257	0.110–0.597

Only variables with *p* < 0.05 in univariate analysis were entered into the multiple logistic regression models.

Backward stepwise selection (entry *p* = 0.05, removal *p* = 0.10) was used to obtain the final model.

OR = odds ratio; CI = confidence interval.

In the univariate logistic regression analysis of depression, participants aged 46–69 years had a higher risk of depression than those aged 18–45 years (OR 0.324, 95% CI: 0.105–0.997; *p* = 0.049). In addition, the front-line of working (OR 2.241, 95%CI: 1.147–4.380; *p* = 0.018) and poor sleep (OR 8.421, 95%CI: 4.122–17.202; *p* < 0.001) contributed to a higher risk of depression. However, a medium level of professional identity was associated with a lower risk of depression than a low level of professional identity (OR0.191, 95% CI: 0.086–0.423; *p* < 0.001). In Table 5, the results of multiple analyses suggested that the PSQI score (OR 6.838, 95.0%CI: 3.260–14.324; *p* < 0.001) and a medium level of professional identity (OR 0.257, 95%CI: 0.110–0.597; *p* = 0.002) were independent factors for depression. The association between professional identity and depression was attenuated after adjustment for sleep quality (PSQI), suggesting that sleep quality may partially mediate the observed bivariate association between professional identity and depression.

## Discussion

To our knowledge, this is one of relatively few quantitative studies have examined the relationship between professional identity and nurses’ mental health specifically in the aftermath of the COVID-19 public health emergency. Our findings add to this literature by examining cross-sectional associations between professional identity, sleep quality and symptoms of anxiety and depression in nurses surveyed in February 2023. Firstly, the characteristics of the anxious group and the non-anxious group were analyzed, reporting professional title, marital status, working years, external support experience, working on the clinical frontline, PSQI score, and professional identity level had increased the risk of anxiety. In addition, age distribution, working on the clinical frontline, PSQI score, and professional identity level will increase the risk of depression in the depressed and non-depressed groups. Nurses working on the clinical frontline may be at higher risk for anxiety and depression. A previous study reported that nurses working in SARS wards were at higher risk of depression and anxiety than nurses working in non-SARS wards (25), which is consistent with our study. Interestingly, the prevalence rates of anxiety and depression after experiencing the COVID-19 public health emergency in this study were 53.8 and 46.2%, respectively, while results from a study reported that the prevalence rates of anxiety and depression were 46 and 40%, respectively (26). Infected nurses may be more concerned about prognosis than about the possibility of infection. Managers should assess the mental health status of nurses and take more timely measures to reduce the occurrence of anxiety and depression. To identify psychological distress early, voluntary and confidential mental health screenings could be implemented. In addition, psychological counseling resources and peer support groups should be made readily available.

The findings revealed that nurses’ professional identity was negatively correlated with anxiety and depression. This aligns with Social Identity Theory, which posits that individuals derive a sense of self-esteem and emotional stability from their group membership. A strong identification with one’s professional group—such as being a nurse—may buffer psychological distress by reinforcing personal value and purpose during times of crisis. Additionally, according to Professional Identity Formation Theory, the development and reinforcement of a stable professional identity over time can enhance psychological resilience. The loss of statistical significance of professional identity in the fully adjusted depression model likely reflects partial overlap between sleep disturbance and professional identity as predictors of depressive symptoms. In our cross-sectional data PSQI showed a particularly strong association with depression and including PSQI in multivariable models attenuated the professional identity coefficient. This attenuation may reflect mediation (sleep as a pathway), shared variance (overlapping constructs), or both; longitudinal mediation analysis is required to disentangle these possibilities. We therefore interpret the depression model results cautiously. Nurses who view their role positively and consistently are less likely to internalize stress or uncertainty, thereby experiencing lower levels of anxiety and depression. Therefore, more attention should be given to identifying nurses’ professional identity.

The results of this study also showed that the mean score of professional identity was 105.25 ± 20.32. Nurses in the ICU have a lower level of professional identity than those in other departments. The phenomenon may be attributed to the critical patients in the ICU with high mortality rates, although they have received the best medical care. However, this finding should be interpreted with caution, as the cross-sectional design does not allow causal inference, and the difference may also reflect contextual factors such as staffing levels or workplace culture. Therefore, the departmental variation observed in this study should be considered exploratory and warrants further investigation in longitudinal or qualitative research.

The results of this study showed that nurses had a medium level of professional identity after experiencing the COVID-19 public health emergency, which is consistent with Liu’s study but lower than Li’s study (21). The mean scores of the five professional identity dimensions were higher than those in Liu’s study, especially in professional identity evaluation. This may be because under the careful care of nurses, the prognosis of patients has greatly improved, which greatly enhanced the professional identity of nurses. During the COVID-19 public health emergency, nurses’ professional identity has been improved. Therefore, active and effective measures were suggested to maintain the high professional identity of nurses. However, due to the heavy workload, deteriorating working environment, and worry about the poor prognosis, nurses’ professional identity had relatively declined at this stage compared with the beginning of the COVID-19 public health emergency. In addition, due to the strong support from government, populations and society associations, the professional identity of medical workers has been greatly enhanced.

This study also found that nurses who were married, had worked more than 10 years, or had an intermediate job title were more likely to be anxious than those who were single, had worked 1–5 years, or had junior or low job titles. This may be explained by married nurses needing to care more about their families, and senior nurses and nurses with high professional titles usually do not engage in front-line clinical work in China. After the COVID-19 public health emergency, many of their operating skills were not proficient, and their ability to accept new things was relatively lower than that of newly recruited nurses. At the same time, professional titles and positions are often related, and nurses with high professional titles often invested more energy and time in management in China.

The results showed that medical assistance, PSQI score, and level of professional identity were independent factors of the anxiety groups. Nurses who had medical assistance of the COVID-19 public health emergency were more likely to have worse anxiety status than those who did not. Medical assistance in this study denotes deployment of nursing staff to assist other regions or hospitals during the COVID-19 response (for example, nurses who were sent to support facilities in harder-hit locations). One possible reason is that most of them had direct contact with patients during the COVID-19 public health emergency. A variety of studies have shown that it may induce mental health problems (27), and medical staff may have higher rate of mental health problems than residents (28). In addition, many studies have reported that health workers had a higher risk of poor sleep quality than others (29). Additionally, only the PSQI score was an independent factor for depression. Some studies have shown that anxiety and depression are both significantly associated with poor sleep quality (30–32). Beyond rotation systems, hospitals should consider increasing staffing ratios, particularly in high-intensity units such as ICU and emergency departments. Studies have shown that insufficient staffing and high patient acuity significantly contribute to nurse burnout and anxiety (30). Moreover, institutions should provide on-site mental health professionals or hotlines for timely psychological support.

Finally, the observed lower mean professional identity in ICU nurses should be interpreted with caution because the ICU subgroup in our sample was small (n = 9). We have deleted strong inferences from this underpowered subgroup analysis and now present it only as an exploratory observation that requires confirmation in larger, purposefully sampled studies. Recent qualitative work has documented identity disruption, role strain and resilience among nurses during the COVID-19 pandemic which complements our quantitative findings and suggests directions for future mixed-methods work (33). Based on the observed associations, hospitals could consider (1) routine, confidential mental-health screening of nursing staff after major public health events; (2) accessible psychological counseling and peer-support programs; (3) targeted sleep hygiene interventions for staff with poor PSQI scores; and (4) staffing adjustments such as temporary rotation/relief to reduce continuous high-acuity exposure. These are proposed pending evaluation in interventional or longitudinal research.

## Conclusion

Nurses’ professional identity was inversely associated with symptoms of anxiety and depression in this cross-sectional survey conducted after the COVID-19 public health emergency. Because of the cross-sectional design causal inferences cannot be drawn; longitudinal and qualitative studies are needed to clarify causal pathways and to test whether interventions to support professional identity reduce psychological symptoms. In addition, psychometric evaluation of the professional identity scale in post-emergency contexts is recommended.

## Limitations

The limitations of this study should be noted. Because this study was cross-sectionally designed, selection bias cannot completely be ruled out. In addition, compared with our large population nurses in China, the sample size of our study was relatively small. Furthermore, the sample was drawn from four hospitals using convenience sampling, the findings may not be fully generalizable to all Chinese nurses. Future studies using stratified random sampling across regions and hospital levels are recommended.

## Data Availability

The raw data supporting the conclusions of this article will be made available by the authors, without undue reservation.
